# Supplementary material for: The interactome of intact mitochondria by cross-linking mass spectrometry provides evidence for coexisting respiratory supercomplexes
Source: Mol Cell Proteomics. 2017 Dec 8;17(2):216–32. doi: 10.1074/mcp.RA117.000470 (PMC5795388; doi:10.1074/mcp.RA117.000470)
Supplement: Supplemental Data [file supp_RA117.000470_133922_0_supp_23978_fzffwf.zip › spectra_annotation/mito_DR_spectra_annotation/22-1-1-1-7-1.pdf]

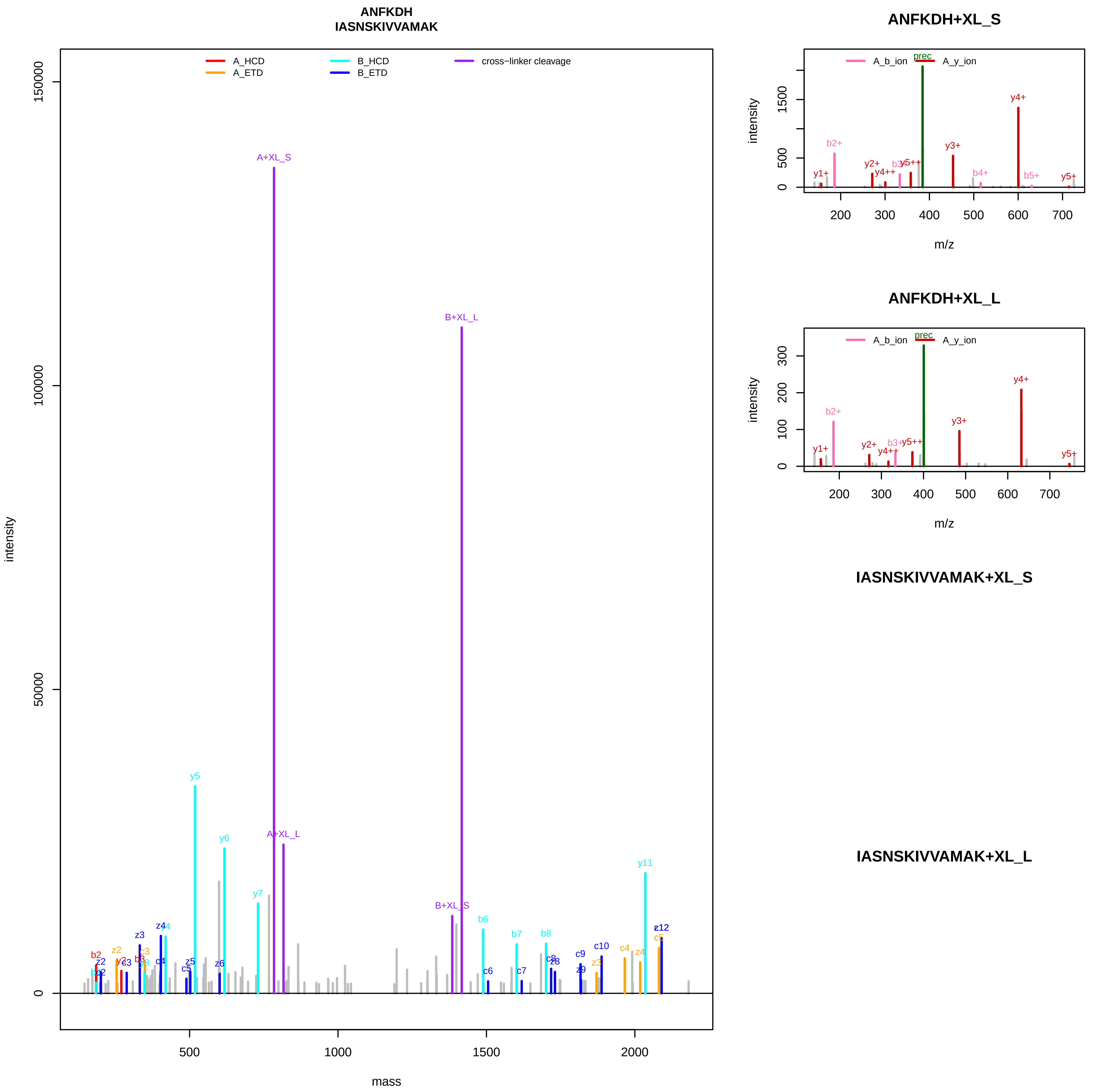
ANFKDH
IASNSKIVVAMAK
A_HCD
A_ETD
B_HCD
B_ETD
cross−linker cleavage
A+XL_S
B+XL_L
A+XL_L
B+XL_S
y5
y6
y7
y11
b6
b7
b8
c4
c5
c6
c7
c8
c9
c10
z2
z3
z4
z5
z6
z8
z9
z12
intensity
mass
0
50000
100000
150000
500
1000
1500
2000
ANFKDH+XL_S
A_b_ion
prec
A_y_ion
y1+
b2+
y2+
y4++
b3+
y5++
y3+
b4+
y4+
b5+
y5+
intensity
m/z
ANFKDH+XL_L
A_b_ion
prec
A_y_ion
y1+
b2+
y2+
y4++
b3+
y5++
y3+
y4+
y5+
intensity
m/z
IASNSKIVVAMAK+XL_S
IASNSKIVVAMAK+XL_L
